# Heavy Metal Accumulation in Rice and Aquatic Plants Used as Human Food: A General Review

**DOI:** 10.3390/toxics9120360

**Published:** 2021-12-20

**Authors:** Mohammad Main Uddin, Mohamed Cassim Mohamed Zakeel, Junaida Shezmin Zavahir, Faiz M. M. T. Marikar, Israt Jahan

**Affiliations:** 1 Institute of Forestry and Environmental Sciences, Faculty of Science, University of Chittagong, Chittagong 4331, Bangladesh; main@cu.ac.bd; 2 School of Biological Sciences, The University of Queensland, St Lucia, Brisbane, QLD 4072, Australia; 3 Department of Plant Sciences, Faculty of Agriculture, Rajarata University of Sri Lanka, Puliyankulama, Anuradhapura 50000, Sri Lanka; 4 Centre for Horticultural Science, Queensland Alliance for Agriculture and Food Innovation, The University of Queensland, Ecosciences Precinct, Dutton Park, Brisbane, QLD 4102, Australia; 5 Australian Centre for Research on Separation Science, School of Chemistry, Monash University, Melbourne, VIC 3800, Australia; shezmin.ismail@gmail.com; 6 Staff Development Centre, General Sir John Kotelawala Defense University, Ratmalana 10390, Sri Lanka; faiz@kdu.ac.lk; 7 Department of Environmental Science, Faculty of Science and Technology, Bangladesh University of Professionals, Mirpur, Dhaka 1216, Bangladesh; israt.env@gmail.com

**Keywords:** heavy metals, aquatic plants, food chain, health risks, ecological challenge

## Abstract

Aquatic ecosystems are contaminated with heavy metals by natural and anthropogenic sources. Whilst some heavy metals are necessary for plants as micronutrients, others can be toxic to plants and humans even in trace concentrations. Among heavy metals, cadmium (Cd), arsenic (As), chromium (Cr), lead (Pb), and mercury (Hg) cause significant damage to aquatic ecosystems and can invariably affect human health. Rice, a staple diet of many nations, and other aquatic plants used as vegetables in many countries, can bioaccumulate heavy metals when they grow in contaminated aquatic environments. These metals can enter the human body through food chains, and the presence of heavy metals in food can lead to numerous human health consequences. Heavy metals in aquatic plants can affect plant physicochemical functions, growth, and crop yield. Various mitigation strategies are being continuously explored to avoid heavy metals entering aquatic ecosystems. Understanding the levels of heavy metals in rice and aquatic plants grown for food in contaminated aquatic environments is important. Further, it is imperative to adopt sustainable management approaches and mitigation mechanisms. Although narrowly focused reviews exist, this article provides novel information for improving our understanding about heavy metal accumulation in rice and aquatic plants, addressing the gaps in literature.

## 1. Introduction

The world’s ever-increasing population places great importance on the availability of sufficient food sources. This has resulted in an increased demand for global food production to fulfil the needs of the growing population, leading to various facades of environmental pollution. Of the various pollutants contributing to the damage to the environment, heavy metals are well recognised, particularly due to their persistence in the environment, toxicity, and bio-accumulative nature, leading to unfavourable repercussions on human health and the ecosystem [1,2].

Heavy metals generally refer to elements that are metals and metalloids which have an atomic weight between 63.5 and 200.6, specific gravity greater than 5.0, and atomic density greater than 4 g cm^−1^ [3]. In addition to natural sources of heavy metals that originate from weathering of metal-bearing rocks, volcanic eruptions, and atmospheric depositions, anthropogenic activities including mining, leakage and emissions from industries, use of agrochemicals including fertilizers, and application of sewage sludge to crop lands are the key sources of heavy metal accumulation in soil and water ecosystems [2]. Heavy metal accumulation can further be defined as an amalgamation of heavy metal elements to the ecosystem, particularly to the aquatic ecosystem [4].

Rice and an array of aquatic plants such as water chestnut (*Trapa* spp.), water spinach (*Ipomoea aquatica*), watercress (*Nasturtium officinale*), taro (*Colocasia esculenta*), and lotus (*Nelumbo nucifera*) are important sources of food, particularly in many Asian countries as well as in West and Central African regions [5]. These plants accumulate heavy metals causing various issues to human health, the environment, and ecosystems [6,7].

Several studies and reviews have been conducted on various aspects of heavy metal accumulations in plants, their remediation, as well as their impact on plant, human, and animal health. There is however a scarcity of recent reviews or gathered information to improve our understanding in connection with heavy metal accumulations in rice and aquatic plants that are used as human food in an increasing global trend. Therefore, this review article highlights the sources of heavy metals for plant bioaccumulation and the mechanisms of heavy metal accumulation in rice and aquatic plants. We further summarise the various health impacts to humans and animals caused by heavy metal accumulation in rice and aquatic plants. In addition, emphasis is placed on the various ecological and environmental challenges of heavy metal accumulation and methods to mitigate them for environmental sustainability. Finally, we also provide a glimpse of the research gap in this sphere that needs to be addressed by future research.

## 2. Importance of Rice and Aquatic Plants as Human Food

Establishing civilisations adjacent to sources of water has always been of great importance to support the cultivation of plants for dietary needs. Of the three main aquatic ecosystem types—namely, marine (seas and oceans), freshwater (rivers, streams, and wetlands), and estuarine (sea water mixed with freshwater)—freshwater systems by far are the most routinely used for fostering cultivation.

Plants found in such aquatic ecosystems are generally referred to as *aquatic plants* or ‘hydrophytes’ and are broadly defined as “macrophytes that grow in water or on a substrate that is at least periodically deficient in oxygen as a result of excessive water content; plants typically found in wetlands and other aquatic habitats” [8]. These plants can be free-floating, floating rooter, submerged, or emergent, and they have adapted to their habitat and the anaerobic atmosphere via a range of adaptations and responses. These include morphological traits (e.g., shallow loosely packed root systems, succulent leaves, hollow stems, enlarged cells for increased oxygen diffusion, adventitious roots, etc.) [9], physiological traits (e.g., metabolic adaptations, increased ethylene production, anaerobic respiration, reoxidation of NADH, etc.) [10,11], and a variety of other traits including seed germination under water, growth dormancy during flooding, viviparous seeds, and root regeneration [8,12,13]. Amongst the wide range of aquatic plants, some plants hold significance due to their long tradition of being used for human consumption. These are categorised as *edible marine plants* (such as macro algae and seaweeds) and *edible freshwater plants*, the latter of which are cultivated to meet human demand or collected from the wild [5]. Whilst a limited number of aquatic plants are used as human food, a large consortium of aquatic plants is used for medicinal needs, mainly based on traditional knowledge localised to specific regions of the world, which include tropical and subtropical Asia, Africa, Central America, Australia, and some parts of Europe and America [5].

Of all aquatic food sources, rice (*Oryza sativa*), a cereal crop belonging to the family Poaceae, takes prominence as a major agronomic species and is the staple diet of over half the world’s population [14]. Approximately 92% of the world’s rice—the second largest produced cereal in the world—is grown in India, China, and the rest of Asia, where it is the main source of energy for its consumers—a number estimated to reach 4.6 billion people in 2025 [15]. In addition, a number of other aquatic plants have been used by humans from time immemorial in all continents of the world, with differences in the way the plant is used depending on the local cuisine. Table 1 presents some commonly used aquatic plants used as food.

The edible part of aquatic plant food varies from plant to plant and may comprise of a single part or different parts, depending on the development stage of the plant as set out in Table 1, and may include roots, rhizomes, corms, stems, leaves, flowers, grains, pollen, inflorescence, or even the whole plant. For example, the main part of the rice (*Oryza sativa*) plant consumed is the grain, whereas it is the leaves of the water spinach (*Ipomoea aquatica*) plant. In contrast, many parts of *Typha* spp., a plant in the family *Typhaceae* (Cattail), are edible, which include starchy rhizome, green flower spike, and shoots [25].

In addition to these major species, a variety of minor semi-aquatic and aquatic plant parts including roots, leaves, flowers, rhizomes, and fruits are also used as food. Examples of these include alligator weed (*Alternanthera philoxeroides*), Bengal cardamom (*Amomum aromaticum*), ghechu (*Aponogeton natans*), Indian pennywort, gotukola (*Centella asiatica*), water hyacinth (*Eichhornia crassipes*), yellow burrhead (*Limnocharis flava*), Australian water lily (*Nymphaea gigantea*), broadleaf arrowhead (*Saggitaria latifolia*), tape grass (*Vallisnaria natans*), rootless duckweed (*Wolffia arrhizal*), sweet flag (*Acorus calamus*), sessile joyweed (*Alternanthera sessilis*), water hawthorn (*Aponogeton distachyos*), Madagascar water lettuce (*Aponogeton ulvaceus*), water sprite (*Ceratopteris thalictroides*), Ceylon hydrolea (*Hydrolea zeylanica*), rice paddy herb (*Limnophila aromatica*), water pepper (*Polygonium hydropiper*), arrowhead (*Sagittaria sagittifolia*), wasabi (*Wasabia japonicum*), and arrow leaf elephant ear (*Xanthosoma sagittifolium*) [5].

## 3. Heavy Metals Present/Accumulated in Rice and Aquatic Plants

Water bodies around the world may get enriched with nutrient or contaminated with unfavourable chemicals from either natural processes or runoff from anthropogenic activities. The diverse range of chemicals used in industrial or agricultural applications can make their way to wastewater sludge, sewage sludge, and aquatic environments downstream of the industry, which can result from regulatory oversights of the manufacture, transport, and disposal of such chemicals [17]. The need to increase food production has also resulted in cultivations being established in polluted land, polluted water being sourced for irrigation, and indiscriminate use of chemical fertilisers and pesticides in efforts to increase crop yield [27,28,29]. Such activities have a direct effect on aquatic food sources in their vicinity.

Aquatic macrophytes play a pivotal role in the nutrient recycling and aerobic or anaerobic conditions of the water bodies they are present in [30]. They have the remarkable capability of absorbing nutrients and pollutants, accumulating them in their tissues, and growing in unfavourable conditions [31]. Heavy metals are one of the most serious offenders of polluting aquatic systems, mainly due to their toxicity, persistence in the environment, and incorporation into food chains [32].

The direct use of plants to remove pollutants by uptake/absorption and bring about remediation is referred to as *phytoremediation*. This can be executed by aquatic plants in the form of *phytoaccumulation* (contaminant accumulation in roots or shoots) or *rhizofiltration* (contaminant uptake by roots in water) [33,34,35]. Some aquatic food sources have shown to be reliable sources of treating contaminated land. For example, *Eleocharis dulcis* (Chinese water chestnut) is used to treat uranium mine runoff in Australia [36], and *Neptunia oleracea* (water mimosa) has been identified as a feasible phytoremediator to clean aquatic systems contaminated with arsenic (As) [20]. Despite the advantages of being able to use aquatic plants to remove heavy metals, the disadvantage stands in the high probability of it being harmful to humans by entering food chains or direct consumption.

As the most abundantly prevalent aquatic food source, the accumulation of heavy metals on rice plants can have widespread repercussions. Due to their ability to adapt to waterlogged or submerged conditions by forming special air channels called aerenchyma which allow O_2_ transport to submerged tissues, contamination of irrigation water can lead to accumulation of heavy metal in rice plants. Sharma et al. [37] summarises some recent studies from various locations worldwide on the contaminations of rice grains with potentially toxic elements and exemplifies a range of elements including lead (Pb), cadmium (Cd), chromium (Cr), iron (Fe), zinc (Zn), arsenic (As), uranium (U), thorium (Th), copper (Cu), nickel (Ni), molybdenum (Mo), manganese (Mn), barium (Ba), and antimony (Sb).

In most nations, rapid economic development and urbanisation can lead to attempts by people to combine traditional cultivation methods with urbanised practices, often using unsuitable urban environments for cultivating crops. Cultivation of aquatic plants for human consumption in water bodies that are contaminated with pollutants and heavy metals can lead to accumulation of metals to various degrees within the plants. *Ipomoea aquatica* (water spinach) is a widely consumed vegetable that is rich in vitamins A, C, and iron and commonly cultivated in tropical and subtropical countries. A sampling of nine sites in the greater Bangkok region in Thailand revealed accumulation of the heavy metals Hg, Pb, and Cd in *I. aquatica*, with some sites having Hg concentrations of up to 1.440 μg kg^−1^ dry weight [38].

Heavy metal contamination in unmanaged water bodies situated in rural areas, and subsequent bioaccumulation in associated aquatic food sources, may not be fully known unless assessed. *Nelumbo nucifera* (wild lotus) is a plant popular in Chinese food and medicine with the entire root, leaf, fruit, and flower being used. A study of *Nelumbo nucifera* plants in an unmanaged pond near Yichang City in China revealed that Cd and As levels exceeded the national food standard with heavy metal concentrations of the edible tuber’s peel, 1.3–9.0 times higher than that in the inner flesh [39].

Some aquatic plants tend to bioaccumulate metals depending on their initial concentration in waters. In such instances, the types of metals accumulated may vary depending on the plant species, as seen in *Nasturtium officinale* (watercress) which tend to accumulate Cd, Cr, and Co in different concentrations [40]. In some instances, the accumulation of heavy metals in plants can also affect aquaponic systems—a bio-integrated system where fish and plants live in a symbiotic environment through the coupling of aquaculture and hydroponics [41]. A rice–fish system has shown that the accumulation of Cd in rice increased to 5.86 mg kg^−1^, which is well beyond the threshold value (Table 2) with the increase in the amount of Cd in water [42].

Whilst some heavy metals such as Fe, Zn, Cu, and Mn become toxic to humans at higher concentrations, metals such as mercury Hg, Cd, As, and Pb are toxic even at trace levels [43]. The direct and indirect detrimental effects of this to human health are manifold and are discussed in detail in a latter section.

## 4. Mechanisms of Heavy Metal Accumulation

It is imperative to understand the mechanism of heavy metal bioaccumulation in aquatic plants used for food, regardless of whether the aim of a study is to assess the presence of heavy metals in aquatic food or whether it is to study the feasibility of a plant for phytoremediation of contaminated water resources. Heavy metal accumulation in plants is affected by several factors, such as the plant’s growth stage, variation in plant species, elemental absorption, tolerance levels to different contaminants, growth rate, and biomass [40]. Although the accumulation capability of aquatic plants is lower than that of terrestrial plants, they can still accumulate a large quantity of metals when they have dense root systems which increases the surface area for absorption of metals [44].

Plant species used for phytoremediation should have the features of being native, having quick growth rates, high biomass yield, extensive root systems, tolerance to high salinity and pH, and various habitat adaptations, as well as the ability to accumulate the absorbed pollutants [45,46]. Unfortunately, most of these features are often the same characteristics displayed by aquatic plants used as food—the very reason they tend to absorb heavy metals easily. Phytoremediation brought about by aquatic plants is mainly through rhizofiltration or phytofiltration, where organic, inorganic, and heavy metal contaminants are taken up by absorption or adsorption and accumulate in the root or aerial parts.

The mechanism of uptake of heavy metals by aquatic plants can differ based on the type of plant and the level of metal-polluted water. The ability of plants to carry out elemental accumulation from the substrate is referred to as the bioconcentration factor (BCF), which is a deciding factor in judging their bioaccumulation capabilities. As such, those with high BCF are referred to as hyperaccumulators. The BCF value of As, Cd, and Pb in brown rice have been in the range of 0.001–0.224, 0.001–2.434, and 0.001–0.048, respectively [47]. The bioaccumulation coefficient is the ratio between the metal concentration in dried tissue of the plant and that in the surrounding medium. This coefficient can vary between different metals and range from several hundred for species such as As and up to 10,000 for cationic species such as Cu and Pb [48]. Metals which are taken up by plant tissues may leak back into the surrounding medium, and hence the net uptake of metals by a plant—which in turn is influenced by external and internal factor—depends both on the uptake as well as the leakage. The metal uptake in aquatic plants is also influenced by a range of biotic and abiotic factors which include temperature, pH, and ionic populations of the aqueous systems [49].

Metals present in the contaminated water—mainly as positive ions—bind to negatively charged binding sites on the plant cell wall [49]. Depending on the affinity of metals to these sites, there may be a hard and non-exchangeable bond or a looser and more exchangeable bond [49]. This binding creates a gradient across the membrane, promoting metal transport into the cell [50]. This uptake increases with external metal concentration but not necessarily with a linear correlation [49]. With time, as the metal concentration in tissues increase, leading to a saturation, a subsequent decrease in effective uptake is seen. This may also occur due to the toxic effects caused by metals, such as oxidative stress, which can be caused by Cu, Cd, and Zn [51].

**Figure 1:**
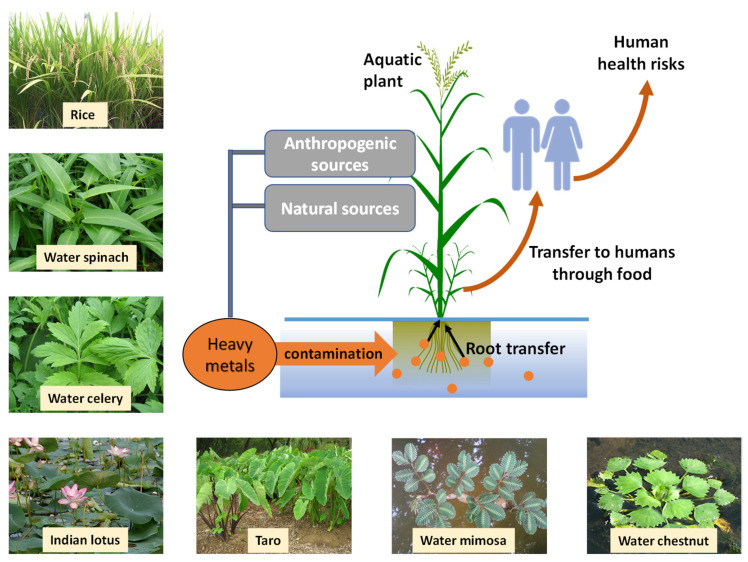
Pathway of heavy metal transfer from original sources to humans. Some common aquatic plants used for human food is inset in the image.

Upon entering the plant roots, metal ions may be stored in the root or transported to the shoot—most probably through the xylem, with some suggestions that the phloem could contribute to this as well [52]. The transport of metals within the xylem or phloem can be facilitated by binding to organic acids, phytochelatins, or metallothioneines [52]. The heavy metals thus accumulated through the roots within the polluted water may deposit in various parts of the aquatic plant, posing detrimental consequences when the plant is used as a food source. Figure 1 depicts a schematic representation of the uptake and distribution of heavy metals in aquatic plants as well as some images of some common aquatic plant foods.

## 5. Sources of Heavy Metals for Bioaccumulation

Waterways contribute significantly to the economy of human populations which rely on them. Pollution of such waterways due to a wide range of reasons such as rapid pace of urbanisation, industrialisation, and change in the way land is used can be a substantial threat to the biodiversity and can subsequently have an effect on the various food sources derived from it. Of the various pollutants, heavy metals play a key role in the disturbance of aquatic ecosystems due to their long biological half-life, non-biodegradability, and environmental persistence, thus contributing to devastating economic, social, and environmental consequences [53]. Some heavy metals have been seen to migrate from one place to another, leading to a change in the landscape as well as a high density in some waterways, and hence knowledge of how its concentration will affect the quality of the humans, plants, and animals is constantly being investigated. Contamination of aquatic and terrestrial ecosystems with toxic heavy metals is an environmental problem of public health concern.

Pollutants present in effluents are categorised as organic and inorganic pollutants—with each category having different ranges of toxic levels. Of these, inorganic pollutants such as heavy metals are of major concern due to their specific oxidation–reduction characteristics, complex forming capability, and solubility [54]. These have a higher density than most other low atomic weight metals and toxicity even at very low levels. Pollution of aquatic ecosystems can render unexpected contaminants in crops dependent on its water and threaten both food security and human health, which is a global concern in an era of diverse and emerging food security issues [55]. This has a direct effect on aquatic plants which are present in polluted waterways due to the respective heavy metals being accumulated in the edible plant parts, such as rice grains and tubers of plants grown in aquatic environments. The seriousness of heavy metal contamination in affected waterways has one of its biggest effects on the harm it poses to living biota. This is due to its ability to be biomagnified through food chains, leading to a strong presence in tradable food crops [37,56].

Sources of entry of heavy metals to the environment may be categorised into two broad groups as *lithogenic sources* and *anthropogenic sources*. Lithogenic sources include weathering of soil minerals, volcanogenic particles, windblown dust, sea salt, and forest wildfires. The large range of anthropogenic sources include, but are not limited to, industrial activities and the waste they generate (battery production, metal products, metal smelting, cable coating industries, etc.), sewage sludge, brick kilns, agrochemicals (pesticides and fertilizers), wastewater irrigation, fossil fuel emission, power plants (coal combustion), etc. Furthermore, mining and industrial processing for extraction of mineral resources and their subsequent applications for industrial, agricultural, and economic development has led to an increase in the mobilization of these elements in the environment and disturbance of their biogeochemical cycles. In the recent past, electronic waste dumps, which are a consequence of manufacturing high numbers of electronic equipment, have been a source of much concern to human and ecological risks due to the presence of excessive amounts of toxic heavy metals [57].

Upon entry to plants through these various sources, the effects that heavy metals have on plants can be multitude, especially since heavy metals can be divided into two groups based on their interactions with plants: (i) elements which are essential for plant growth such as Fe, Mo, Ni, and Zn, which can become toxic when their concentrations exceed specific threshold levels and (ii) elements which are non-essential to plants such as Cd, Hg, Pb, and As. As such the greatest hidden risks may be posed by those heavy metals which are toxic to plants at very low concentrations and yet may accumulate in plant tissues in higher concentrations whilst showing few changes in reduction yield or visible symptoms [58,59].

## 6. Heavy Metals in Food Chain from Rice and Aquatic Plants to Humans

As outlined above, of the multitude of elements absorbed by plants, some elements are considered to be essential because the plants need such elements as part of their life cycle. However, being persistent pollutants, these heavy metals accumulate in the environment and consequently contaminate the food chains [60,61]. Since the quantities of the elements in water can vary from place to place, the amounts taken up by aquatic plants and retained in their tissues can also show considerable variations [59].

Metals such as Fe, Mn, Mo, Cu, Zn, and Ni are required by plants in minute quantities and are referred to as ‘*micronutrients*’, whilst metals such as aluminium (Al), silver (Ag), gold (Au), and cobalt (Co) have a stimulatory effect on plant growth. On the other hand, metals such as As, Cd, Cr, Hg, and Pb have no known biological function yet can be absorbed by plants and be toxic even at low concentrations [62]. Previous literature has reported that bioaccumulation of heavy metals such as As, Cd, Co, Cr, Hg, Ni, and Pb in rice, along with some aquatic plants such as water spinach, Indian lotus, and watercress, have been detected as being beyond the permissible limit when compared with the allowable limits of the World Health Organisation (WHO) and the Food and Agriculture Organisation (FAO) of the United Nations [63,64] (Table 2). This suggests the need for further research to overcome these challenges for human health safety and environmental sustainability.

Plants can be divided into three main categories, depending on their ability to cope with heavy metals in the medium they grow in [73].

1. Indicator plants—plants which are usually sensitive to heavy metals. These can be used as indicators as for the presence of metal in the substrate they have grown in.
2. Excluders—these plants can tolerate heavy metals in the substrate up to a threshold concentration. This is achieved by preventing the accumulation of the heavy metal in the cell by either blocking the uptake in roots or by energy dependent efflux pumps. Most metal (hyper) tolerant plants are categorised into this group.
3. Hyperaccumulators—in addition to the ability to tolerate high concentrations of specific elements, these plants can actively take them up and accumulate them in their aerial parts. Often these plants have specific mechanisms to avoid poisoning themselves by the accumulated metals.

The quantity of heavy metals accumulated in aquatic plants also depends on various physicochemical factors, such as the bioavailability in water, absorption rate through the plant membrane, stability of the metal in the biotic and abiotic environment, distribution in the various plant tissues, and the ability to form deposits in tissues [74]. The concentration of the heavy metal is comparatively greater in higher links of a food chain than their respective concentration in the lower links [75]. Thus, the heavy metals which may have bioaccumulated in aquatic plant foods make their way to humans along the food chain [75]; this may be through direct ingestion of plant product such as rice, or via consumption of animals who may have consumed the polluted aquatic plant. Once in humans, heavy metal accumulation often takes place in some target organs which serve as deposits of these metal elements. These are however not seen to be subsequently excreted in mothers’ milk, unlike the other group of persistence chemicals such as aromatic organochlorine compounds, including dichlorodiphenyltrichloroethane (DDT), polychlorinated biphenyl (PCB), and dioxins [76].

Aquatic ecosystems in which aquatic plants for human food are grown often have other human food organisms within them, such as fish. In inland regions of any part of the world, human exposure to Hg comes more from rice than fish [77]. Often Asiatic nations may be affected more from human exposure to Hg in wastewater compared with other parts of the world due to their dependence on rice as a staple diet. This is because paddy lands are transient wetlands that are regularly flooded, facilitating the methylation processes that make Hg more biologically available in agricultural products [78].

Certain heavy metals found in wastewater are non-threshold toxins which may render toxic effects even at low concentrations. These include As, Cd, Cr, Pb, and Hg, all of which cause risks for human health upon direct ingestion or build up through food chains [79]. These can be found in different forms in the environment, as outlined in Table 3 [79].

## 7. Human Health Risk Associated with Heavy Metal Accumulation in Food

The accumulation of heavy metals in the human body has been demonstrated to have adverse effects on human health. Heavy metals that possibly cause adverse effects include As, Al, Fe, Cd, and Hg [80]. These metals can enter the body through various ways, such as skin or inhalation routes or intake of heavy metals through contaminated drinking water and food. Heavy metals can also react with certain compounds in the body, such as oxygen and chloride, exerting their own toxic effects [81]. Persistent exposure to heavy metals can lead to an imbalance in the body when heavy metals accumulate in the body and are used as substitutes for essential elements. Examples of heavy metals replacing essential elements of the human body include calcium replaced by lead, zinc by cadmium, and most trace elements by aluminium [82].

Most heavy metals are dangerous to humans due to their non-biodegradable nature and ability to accumulate in human tissues. Often even very low amounts of metals can cause disruption or damage to vital body functions due to the lack of suitable mechanisms to eliminate such metals from the body [83]. Humans exposed to heavy metal-polluted food may display a range of symptoms and diseases both in the short term as well as the long term [83,84]. These may affect various human body systems such as pulmonary; renal; gastrointestinal; skin; neurological; etc. systems and may result in conditions such as cardiovascular problems; depression; hematic, gastrointestinal, and renal failure; neurological damage; osteoporosis; tubular and glomerular disfunction; and various cancers [84,85,86,87,88]. Furthermore, a lack of immunological defences may be seen due to the depletion of essential nutrients in the body [71]. Heavy metal poisoning has been seen to have adverse effects in infants, children, and adolescents, which may result in developmental challenges and a decrease in intelligence quotients [89].

Most countries have imposed regulations for maximum levels (MLs) of toxic elements allowed in human food to prevent the consumption of food poisoned by heavy metals. It is not uncommon to see instances where the levels of toxic heavy metals in an aquatic crop exceed this allowable limit. For example, in a study conducted on various rice varieties in Nigeria, it was seen that Pb concentrations exceeded the recommended tolerable weekly intake [71]. Here it was seen that an average of 10.6 μg dL^−1^ of Pb was seen in children 1–6 years old, which exceeded the maximum allowed limit of 10 μg dL^−1^ recommended by the Centre of Disease Control (CDC) [71].

Ongoing investigations and studies in different parts of the world have revealed the links that heavy metals ingested through food have with various diseases of unknown aetiology. The aetiological significance of agricultural factors in relevance to Parkinson’s disease revealed that environmental substances, such as heavy metals which can initiate oxidant damage, could play a role in the disease’s aetiology [90].

The results of human exposure to toxic heavy metals can be multifaceted, and these complexities can affect different internal and external organs in humans in both an acute and chronic manner. The pollution of waterbodies can have a direct influence on the presence of heavy metals in aquatic plants and their entry to humans who consume such aquatic plants. For example, consumption of contaminated rice can cause a range of diseases such as cancer of the lung, bladder, and skin through arsenic-contaminated rice and neurotoxicological effects by lead- and mercury-contaminated rice [62,91,92,93,94]. Exposure to toxic heavy metals from contaminated aquatic ecosystems and aquatic plant foods leads to several detrimental effects to human health (Table 4).

## 8. Ecological and Environmental Challenges Due to Heavy Metal Accumulation

Heavy metal accumulation in plants or soils has been a major concern for the last few decades, mainly due to increased industrialisation and urbanisation [4,105]. The accumulation of heavy metals in plants may originate from lithogenic or anthropogenic sources, threatening the entire ecosystem and causing damage to the function and structure of the surrounding environment [106,107].

The damage caused by such heavy metal accumulations can be to both plants as well as animals and humans that depend on the contaminated ecosystem or plants grown on the contaminated aquatic ecosystem [4,108,109]. For example, Pb deposition in rice and aquatic plants impairs the molecular functions of DNA, leading to negative impacts on food producing plants [110,111]. Furthermore, Cd is a highly mobile element in soil systems and consequently harms important microorganisms, while Cu has a negative impact on soil microbes, and Zn damages the activities of beneficial microbes [4]. Therefore, it has been a major challenge to balance the ecosystem in a sustainable manner and avoid the detrimental effects on human health whilst fulfilling the food needs of a growing population. Some of the major ecological and environmental challenges are briefly discussed below.

Heavy metals toxicity is a problem of increasing significance for ecological, evolutionary, nutritional, and environmental reasons. Increased levels of heavy metals in the growing environment of a plant can lead to reduced photosynthesis, plant biomass, growth, crop yield, and quality and can also affect cell metabolism of various plant organs [112]. Furthermore, a range of physiological activities such as respiration, photosynthesis, protein metabolism, and morphogenesis can also be affected [112]. For example, due to the use of As-contaminated groundwater for rice cultivation, most Southeast Asian nations are under threat of As toxicity [113,114,115]. Exposure of rice plants to As leads to its deposition in the nucleus and damage to photosynthetic machinery, inhibition of germination and seedling growth, decrease in tiller number and biomass, reduction in yield of rice, and subsequent higher accumulation in the rice grain and cooked rice [116].

Two main challenging aspects directly related to heavy metals in aquatic food are the use of pesticides and fertilisers. Due to the rapid advancement of technology and decrease in use of traditional cultivation methods, agricultural ecosystems are greatly affected. This is mainly due to the excessive use of pesticides and many dangerous chemicals containing heavy metals [117,118]. Uncontrolled use of pesticides, which includes herbicides, insecticides, and fungicides, leads to their increased presence in water bodies on which aquatic plant foods depend [119]. Their subsequent biological accumulation in food chains of complex ecosystems leads to high risks for mammals, other living organisms, and humans exposed to these chemicals [119]. Both the direct and indirect impact of pesticides on living organisms results in an imbalance of the nearby ecosystem, as these pesticides can stay in the ecosystem for a long period with carcinogenic effects on human health [4]. Apart from carcinogenic effects, pesticides with heavy metals can contribute to a wide range of diseases, such as asthma, hormonal disturbances, hypersensitivity, congenital disabilities, reduction in birth weight, and even death [120].

Fertilisers consisting of organic and inorganic materials are the main sources of heavy metal accumulation in soil, waterways, and in plants through nutrient uptake processes. For example, phosphorous is one of the commonly used fertilisers that plays a key role in accumulating heavy metals in soils and plants [121]. Rock phosphate, which is one form of phosphate fertilizers, may also contain trace amounts of Cd [122]. Excessive application of fertilisers for long durations leads to accumulation of heavy metals in waterbodies and aquatic plants, which may result in decreased plant growth and food productivity and a further imbalance of the ecosystem.

The use of treated and processed sewage sludges (biosolids) which are generated during the treatment of wastewater is used as an alternative to traditional fertilisers in agriculture [123]. Such biosolids are a rich source of nutrients, especially phosphorus, which is an element essential for plant growth [124]. These sludges however can also contain various pollutants, including heavy metals, which may call for analysis of their ecotoxicological effects on aquatic organisms to ensure harmless use [124].

## 9. Mitigation of Heavy Metal Accumulation in Rice and Aquatic Plants

One of the main principles of preventing food from being contaminated by heavy metals is to ensure the lowest possible levels of contamination in food by implementing good work practices and safe agricultural and irrigation practices [125]. Establishing maximum limit guidelines and legislation and adhering to these guidelines are also greatly beneficial [126]. However, this may not always be practical and feasible in instances where rural communities rely on naturally grown aquatic plants [127,128]. Suitable management of heavy metal-contaminated ecosystems plays a pivotal role in maintaining environmental health and in ecological restoration [46].

Maximum limits set for specific contaminants in food help in protecting public health. The risk associated with exposure to heavy metals in contaminated food is calculated by the daily intake rate (DIR), calculated in μg day^−1^ using Equation (1), where *C* denotes the concentration of heavy metal in the specific plant food (mg kg^−1^ of fresh weight), *IR* is the plant food ingestion rate (g fresh weight per person per day) in the region being investigated, and *BW* is the average adult body weight (kg) [129].


(1) $$DIR\;=\;\frac{C\times IR}{BW}$$

To reduce the impact of heavy metal pollutants in water bodies, removal of the heavy metals via bioremediation is imperative [125,130]. The various physical and chemical remediation approaches are restricted and localized and are mainly applied to wastewater and/or contaminated soils and not the plant life that may reside there [131,132]. Some phytoremediation methods are well explored but might not always be suitable for edible crops and vegetables [131,132].

To move past these issues, biotechnological and nanotechnological approaches can open a path ahead for removing the metals from contaminated vegetables and plants by successfully superseding traditional methods. Certain microorganisms, for example, can respond to heavy metal stress through transport across the cell membrane, entrapment in extracellular capsules, precipitation, biosorption to cell walls, complexation, and redox reactions. Microorganisms can also encounter heavy metal stress using diverse defensive systems, such as compartmentalization, exclusion, formation of complexes, and the synthesis of binding proteins such as metallothioneins (MTs) or phytochelatins (PCs) [133].

More recent approaches also include a new dimension in agriculture referred to as nano-fertilisers, where there is an efficient use of water and fertilisers by plants to render a better managed nutrition system [134]. Here nutrients bound to nano-dimensional adsorbents facilitate a more targeted and slower release of nutrients compared with conventional methods. This in turn reduces the prevalence of excessive heavy metals being released to aquatic ecosystems.

**Figure 2:**
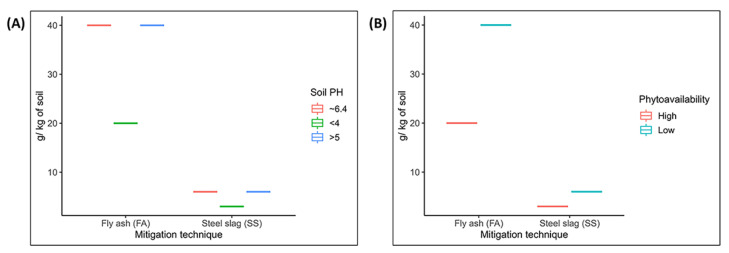
Status of (**A**) soil pH and (**B**) phytoavailability when fly ash (FA) and steel slag (SS) were used in agriculture (rice) soil. Y-axis indicates amount of FA or SS in grams per one kilogram of soil. Data were obtained from Gu et al. [135].

The heavy metals accumulation problem in aquatic plant foods has become an issue requiring urgent attention, and it is necessary to mitigate this raising concern as soon as possible [4]. A pot experiment on heavy metal accumulation in rice revealed that the use of fly ash and steel slag elevated soil pH and reduced the phytoavailability of heavy metals significantly [135] (Figure 2).

Some popular and widely used techniques to mitigate heavy metals accumulation in rice and other aquatic plants are summarised in Table 5.

## 10. Conclusions

With their origin in various natural and anthropogenic sources, heavy metals may contaminate aquatic ecosystems. Use of contaminated water for irrigation of crops can allow direct transport of heavy metals into plants. The implications of heavy metal contamination in aquatic ecosystems are substantial in situations where the water sources in such ecosystems support rice and other aquatic plants intended for human consumption—this is mainly because rice is a staple in the diet of most Asian countries and many other nations worldwide, as well as because of the prevalence of numerous other aquatic plants used as human food in different parts of the world. Amongst all aquatic crops, rice takes a main role in its capacity to absorb Cd and As metals and poses a strong threat to the extensive human population that depends on it.

Aquatic plants can absorb heavy metals through the root systems that transport the metals to edible plant parts, such as leaves, flowers, corms, stems, seeds, etc., with subsequent introduction into the food chain, as confirmed by various reports. Direct ingestion of heavy metal-contaminated aquatic plants and their bioaccumulation in food chains are the main sources of human exposure to toxic heavy metals from aquatic food plants. This exposure can lead to various chronic and acute diseases and health consequences, such as kidney damage, various cancers, endocrine disruptions, neurological effects, immunological disturbances, developmental issues, etc.

Whilst plants use many heavy metals as micronutrients in low concentrations to fulfil their nutritional and physiological needs, some heavy metals can be phytotoxic, leading to disruptions of developmental and metabolic processes, including the plant’s physiology, photosynthesis, morphology, cell structure, and nutrient balance. These disruptions can lead to decreased growth and yield of food crops as well as ecological imbalance. An array of methods including newer technologies that are being constantly explored have been used over the years to mitigate the deleterious effects caused by heavy metal contamination of aquatic ecosystems that are used for crop production. Progress has been made in phytoremediation of contaminated water bodies, nanotechnological advances for the removal of heavy metals, and genetic modification of plants to tolerate higher levels of accumulated heavy metals. These new approaches should however be accompanied by suitable risk assessment and thorough studies. Special attention should therefore be given to mitigate heavy metal exposure and subsequent accumulation in rice and aquatic plants that are used as human food.

In this review, we focused mainly on various sources of heavy metals for bioaccumulation in rice and aquatic plants that are consumed by humans, the mechanisms of their bioaccumulation, ecological and environmental challenges and health consequences, and ways of mitigating heavy metal contamination of aquatic ecosystems and subsequent bioaccumulation in the plants. However, we did not focus on why plants accumulate heavy metals and their adaptations and coping mechanisms for toxic effects of heavy metal, as well as international policy and regulatory frameworks for addressing the heavy metal contamination of aquatic ecosystems and its subsequent public health concerns. Heavy metal accumulation in aquatic plants remains a field which needs more in-depth analysis and study, together with novel ways of combatting the deleterious effects it has on human and animal health as well as ecosystems at large.

## Figures and Tables

**Table 1:** Types of aquatic plants used as human food and their most commonly consumed part.

Common Name	Scientific Name	Edible Part	Reference
Rice	*Oryza sativa*	Grain	[15]
Wild rice	*Zizania* spp.	Grain	[16]
Water celery/Chinese celery/Japanese parsley	*Oenanthe javanica*	Leaves	[17]
Watercress	*Nasturtium officinale*	Leaves	[18]
Water spinach/Kang kong	*Ipomoea aquatica*	Leaves, stems	[19]
Water mimosa	*Neptunia oleracea*	Leaves	[20]
Chinese water chestnut	*Eleocharis dulcis*	Corm, nuts, leaves	[21]
Water chestnut/Water caltrop	*Trapa natans*	Seeds	[22]
Indian lotus	*Nelumbo nucifera*	Roots, rhizome, stamen, seeds, flowers, leaves	[23]
Taro	*Colocasia esculanta*	Rhizome, leaves, stalks, cormels, inflorescence	[24]
Cattail/Cossack asparagus/Lesser bulrush	*Typha* spp.	Rhizomes, interior of tender shoots, seeds, pollen, flower spike, subterranean baby shoots	[25]
Blue-green algae	*Spirulina* spp.	Biomass	[26]

**Table 2:** Permissible limit for heavy metals in rice and aquatic plants/vegetables with their concentrations in some previous studies.

Heavy Metal	Permissible Limit in Cereals and Vegetables (mg kg^−1^)	Amount Present (mg kg^−1^ Wet Produce)				References
		Rice	Water Spinach	Indian Lotus	Watercress	
As	0.1–0.2	0.09–0.13	-	0.1–1.3 *	2.0 *	[39,65,66,67,68]
Cd	0.05–0.4	0.003–0.06	0.06–1.10 *	0.04–0.09	0.10	[38,39,66,67,68,69,70]
Co	0.01	-	-	-	0.30 *	[69]
Cr	1.3	0.12–0.37	-	1.6–2.2 *	0.34	[39,66,67,68,69]
Cu	20.0	2.6–5.3	-	4.4–7.4		[39,67,68]
Hg	0.03	0.002–0.034 *	1.44 *			[38,66,67,68]
Ni	0.1	0.25 *	-		0.34 *	[38,68,69]
Pb	0.05–0.3	0.01–0.53 *	0.28	0.3–0.8 *	0.86 *	[38,39,66,67,68,69,71]
Zn	60.0	16–36	-	9.8–15.4		[39,67,68,72]

* Values indicate that the concentrations of the heavy metals in some studies have been detected beyond the permissible limit [63,64].

**Table 3:** Forms of arsenic, cadmium, chromium, lead, and mercury found in the environment, leading to build-up in food chains (adapted from [79]).

Element	Main Oxidising States	Natural and Lithogenic Sources	Anthropogenic Sources	Effects on Humans
Arsenic	As(III), As(V)	Weathering of rocks, volcanic eruptions, microbial colonization, As bearing minerals in the lithosphere (e.g., FeAsS, CoAsS, NiAs, AsS, As_2_S, As_2_O_3_)	Fossil fuel combustion, mining, smelting, fertilisers, glass production, chemotherapeutic drug production	Carcinogenic and neurotoxic
Cadmium	Cd(II)	Volcanic activities, weathering, erosion, wildfire, sea salt spray, dust storm, Cd bearing compounds in the lithosphere (e.g., CdS, CdCO_3_, Cu_4_Cd(SO_4_)_2_(OH)_6_.4H_2_O, CdSe)	Ni–Cd batteries, fossil fuel combustions, mining, cement production, plastic stabilisers, coatings industry, phosphate fertiliser	Carcinogenic
Chromium	Cr(III), Cr(VI)	Tectonic and hydrothermal events, in the lithosphere as FeCr_2_O_4_ and PbCrO_4_	Aircraft industry, electroplating, wood preservation, tanning, mining, textile dyes manufacturing, metal corrosion inhibition, and cleaning of glassware	Carcinogenic and Mutagenic
Lead	Pb(II), Pb(IV)	Natural fires, natural deposits, sea salt spray, and volcanic eruptions and over 100 Pb-containing minerals in the lithosphere (e.g., PbS, PbCrO_4_, PbSO_4_, Pb_5_(PO_4_)_3_Cl, PbMn_8_O_16_, PbCO_3_)	Pb–acid battery recycling (PABC), Pb-containing gasoline in petrol, pipes, pesticides, ammunition, electronic wastes, mining, ore processing, pigment in paints, dyes, and ceramic glazes	Neurotoxic
Mercury	Hg, Hg(I), Hg(II)	Weathering of rock, volcanic eruptions, degassing and wildfire. In the lithosphere as metallic form (Hg)(0) (rare) or as HgS, Hg_3_S_2_Cl_2_, HgSb_4_S_8_	Coal combustion, production of non-ferrous and ferrous metals, artisanal and small-scale gold mining (ASGM), cement production, pesticides, and fertilisers production	Neurotoxic

**Table 4:** Human diseases and health conditions caused by exposure to toxic heavy metals found in aquatic plants used as human food.

Heavy Metal	Target Organ	Disease Condition/Clinical Effect	References
Arsenic	Nervous system, skin, pulmonary, gastrointestinal	Nausea, vomiting, multi-organ dysfunction syndrome, long QT syndrome, ‘rice water’ diarrhoea, nasal septum perforation, peripheral neuropathy, encephalopathy, respiratory cancer, skin cancer, prostate cancer, hypopigmentation,	[95,96]
Cadmium	Skeletal, renal, pulmonary	Osteomalacia, proteinuria, glucosuria, emphysema, pneumonitis, inhibition of progesterone and oestradiol, alterations in uterus, ovaries and oviduct, progesterone synthesis of ovaries, endocrine disruption, acting as estrogen in breast cancer, excess risk of cardiovascular mortality	[97,98]
Chromium	Pulmonary, gastrointestinal	Nasal septum perforation, respiratory cancer, ulcers, gastrointestinal haemorrhage, haemolysis, acute renal failure, pulmonary fibrosis, DNA damage	[99,100]
Lead	Nervous system, renal, hematopoietic system, gastrointestinal	Encephalopathy, anaemia, central nervous disorders, peripheral neuropathy, nausea, vomiting, abdominal pain, nephropathy, foot-drop/wrist-drop, damages circulatory system and cardiovascular system	[101,102]
Mercury	Nervous system, renal, gastrointestinal	Proteinuria, fever, vomiting, diarrhea, acute lung injury, nausea, metallic taste, gingivo-stomatitis, tremor, neurasthenia, nephrotic syndrome; hypersensitivity, cough, fever, tremor, malaise, motor neuropathy, gum disease, delusions and hallucinations	[103,104]

**Table 5:** Techniques to mitigate heavy metal accumulation in rice and aquatic plants.

Technique	Purpose/Outcome	References
Chemical washing	Remediation of heavy metals	[136,137]
Electro-remediation	Remediation of heavy metals	[136,137]
Phytoremediation	Remediation of heavy metals	[136,137]
Immobilisation/phytostabilisation	Remediation of heavy metals	[136,137,138]
Amendments (limestone, zeolite)	Stabilisation of heavy metals	[139,140]
Application of fly ash (FA) and steel slag (SS)	Increase soil pH and decrease heavy metal phytoavailability	[135]

## Data Availability

This is a review article, however, data used and/or analysed during the review are available on request from the corresponding author.

## References

[1] Sharma R.K., Agrawal M. (2005). Biological effects of heavy metals: An overview. J. Environ. Biol..

[2] Nagajyoti P.C., Lee K.D., Sreekanth T. (2010). Heavy metals, occurrence and toxicity for plants: A review. Environ. Chem. Lett..

[3] Hawkes S.J. (1997). What is a “heavy metal”?. J. Chem. Educ..

[4] Alengebawy A., Abdelkhalek S.T., Qureshi S.R., Wang M.-Q. (2021). Heavy metals and pesticides toxicity in agricultural soil and plants: Ecological risks and human health implications. Toxics.

[5] Aasim M., Bakhsh A., Sameeullah M., Karataş M., Khawar K.M., Ozturk M., Hakeem K., Ashraf M., Ahmad M. (2018). Aquatic plants as human food. Global Perspectives on Underutilized Crops.

[6] Mishra P., Mishra M., Hashmi M., Varma A. (2018). Risk Assessment of heavy metal contamination in paddy soil, plants, and grains (*Oryza sativa* L.). Environmental Pollution of Paddy Soils.

[7] Mishra S., Bharagava R.N., More N., Yadav A., Zainith S., Mani S., Chowdhary P., Sobti R., Arora N., Kothari R. (2019). Heavy metal contamination: An alarming threat to environment and human health. Environmental Biotechnology: For Sustainable Future.

[8] Tiner R.W. (1991). The concept of a hydrophyte for wetland identification. Bioscience.

[9] Martin J.H., Leonard W.H. (1949). Principles of Field Crop Production.

[10] Drew M., Lynch J.M. (1980). Soil anaerobiosis, microorganisms, and root function. Annu. Rev. Phytopathol..

[11] Magneschi L., Perata P. (2009). Rice germination and seedling growth in the absence of oxygen. Ann. Bot..

[12] Baskin C.C., Baskin J.M. (1998). Seeds: Ecology, Biogeography, and, Evolution of Dormancy and Germination.

[13] Farnsworth E. (2000). The ecology and physiology of viviparous and recalcitrant seeds. Ann. Rev. Ecol. Syst..

[14] Muthayya S., Sugimoto J.D., Montgomery S., Maberly G.F. (2014). An overview of global rice production, supply, trade, and consumption. Ann. N. Y. Acad. Sci..

[15] Gnanamanickam S.S. (2009). Rice and its importance to human life. Biological Control of Rice Diseases, Progress in Biological Control.

[16] Yu X., Chu M., Chu C., Du Y., Shi J., Liu X., Liu Y., Zhang H., Zhang Z., Yan N. (2020). Wild rice (*Zizania* spp.): A review of its nutritional constituents, phytochemicals, antioxidant activities, and health-promoting effects. Food Chem..

[17] Chen W.-C., Huang H.-C., Wang Y.-S., Yen J.-H. (2011). Effect of benzyl butyl phthalate on physiology and proteome characterization of water celery (*Ipomoea aquatica* Forsk.). Ecotoxicol. Environ. Saf..

[18] Klimek-Szczykutowicz M., Szopa A., Ekiert H. (2018). Chemical composition, traditional and professional use in medicine, application in environmental protection, position in food and cosmetics industries, and biotechnological studies of *Nasturtium officinale* (watercress)—A review. Fitoterapia.

[19] Dua T.K., Dewanjee S., Gangopadhyay M., Khanra R., Zia-Ul-Haq M., De Feo V. (2015). Ameliorative effect of water spinach, *Ipomea aquatica* (*Convolvulaceae*), against experimentally induced arsenic toxicity. J. Transl. Med..

[20] Atabaki N., Shaharuddin N.A., Ahmad S.A., Nulit R., Abiri R. (2020). Assessment of Water Mimosa (*Neptunia oleracea* Lour.) Morphological, Physiological, and Removal Efficiency for Phytoremediation of Arsenic-Polluted Water. Plants.

[21] Xiao L., Chen J., Wang X., Bai R., Chen D., Liu J. (2018). Structural and physicochemical properties of chemically modified Chinese water chestnut [*Eleocharis dulcis* (Burm. f.) Trin. ex Hensch] starches. Int. J. Biol. Macromol..

[22] Hummel M., Kiviat E. (2004). Review of world literature on water chestnut with implications for management in North America. J. Aquat. Plant Manag..

[23] Paudel K.R., Panth N. (2015). Phytochemical profile and biological activity of *Nelumbo nucifera*. J. Evid. Based Complementary Altern. Med..

[24] Akwee P., Netondo G., Kataka J., Palapala V.A. (2015). A critical review of the role of taro *Colocasia esculenta* L. (Schott) to food security: A comparative analysis of Kenya and Pacific Island taro germplasm. Sci. Agric..

[25] Fruet A.C., Seito L.N., Rall V.L.M., Di Stasi L.C. (2012). Dietary intervention with narrow-leaved cattail rhizome flour (*Typha angustifolia* L.) prevents intestinal inflammation in the trinitrobenzenesulphonic acid model of rat colitis. BMC Complementary Altern. Med..

[26] Barros de Medeiros V.P., da Costa W.K.A., da Silva R.T., Pimentel T.C., Magnani M. (2021). Microalgae as source of functional ingredients in new-generation foods: Challenges, technological effects, biological activity, and regulatory issues. Crit. Rev. Food Sci. Nutr..

[27] Edwards C.A. (1989). The importance of integration in sustainable agricultural systems. Agric. Ecosyst. Environ..

[28] Novotny V. (1999). Diffuse pollution from agriculture—A worldwide outlook. Water Sci. Technol..

[29] Singh R. (2000). Environmental consequences of agricultural development: A case study from the Green Revolution state of Haryana, India. Agric. Ecosyst. Environ..

[30] Barko J.W., James W.F., Jeppesen E., Søndergaard M., Søndergaard M., Christoffersen K. (1998). Effects of submerged aquatic macrophytes on nutrient dynamics, sedimentation, and resuspension. The Structuring Role of Submerged Macrophytes in Lakes.

[31] Ali S., Abbas Z., Rizwan M., Zaheer I.E., Yavaş İ., Ünay A., Abdel-Daim M.M., Bin-Jumah M., Hasanuzzaman M., Kalderis D. (2020). Application of floating aquatic plants in phytoremediation of heavy metals polluted water: A review. Sustainability.

[32] Proshad R., Kormoker T., Mursheed N., Islam M.M., Bhuyan M.I., Islam M.S., Mithu T.N. (2018). Heavy metal toxicity in agricultural soil due to rapid industrialization in Bangladesh: A review. Int. J. Adv. Geosci..

[33] Sahu Y.K., Deb M.K., Patel K.S., Martín-Ramos P., Towett E.K., Tarkowska-Kukuryk M. (2020). Bioaccumulation of nutrients and toxic elements with macrophytes. J. Hazard. Toxic Radioact. Waste.

[34] Mustafa H.M., Hayder G. (2021). Recent studies on applications of aquatic weed plants in phytoremediation of wastewater: A review article. Ain Shams Eng. J..

[35] Zavahir J.S., Wijepala P.C., Seneviratne G., Seneviratne G., Zavahir J.S. (2021). Role of Microbial Communities in Plant–Microbe Interactions, Metabolic Cooperation, and Self-Sufficiency Leading to Sustainable Agriculture. Role of Microbial Communities for Sustainability. Microorganisms for Sustainability.

[36] Overall R.A., Parry D.L. (2004). The uptake of uranium by *Eleocharis dulcis* (Chinese water chestnut) in the Ranger Uranium Mine constructed wetland filter. Environ. Pollut..

[37] Sharma S., Kaur I., Nagpal A.K. (2021). Contamination of rice crop with potentially toxic elements and associated human health risks—A review. Environ. Sci. Pollut. Res..

[38] Göthberg A., Greger M., Bengtsson B.E. (2002). Accumulation of heavy metals in water spinach (*Ipomoea aquatica*) cultivated in the Bangkok region, Thailand. Environ. Toxicol. Chem. Int. J..

[39] Luo Y., Zhao X., Xu T., Liu H., Li X., Johnson D., Huang Y. (2017). Bioaccumulation of heavy metals in the lotus root of rural ponds in the middle reaches of the Yangtze River. J. Soils Sediments.

[40] Duman F., Leblebici Z., Aksoy A. (2009). Growth and bioaccumulation characteristics of watercress (*Nasturtium officinale* R. BR.) exposed to cadmium, cobalt and chromium. Chem. Speciat. Bioavailab..

[41] Graber A., Junge R. (2009). Aquaponic Systems: Nutrient recycling from fish wastewater by vegetable production. Desalination.

[42] Luo W., Wang D., Xu Z., Liao G., Chen D., Huang X., Wang Y., Yang S., Zhao L., Huang H. (2020). Effects of cadmium pollution on the safety of rice and fish in a rice-fish coculture system. Environ. Int..

[43] Llobet J., Falco G., Casas C., Teixido A., Domingo J. (2003). Concentrations of arsenic, cadmium, mercury, and lead in common foods and estimated daily intake by children, adolescents, adults, and seniors of Catalonia, Spain. J. Agric. Food Chem..

[44] Xing W., Wu H., Hao B., Huang W., Liu G. (2013). Bioaccumulation of heavy metals by submerged macrophytes: Looking for hyperaccumulators in eutrophic lakes. Environ. Sci. Technol..

[45] Valipour A., Ahn Y.-H. (2016). Constructed wetlands as sustainable ecotechnologies in decentralization practices: A review. Environ. Sci. Pollut. Res..

[46] Sundaram L., Rajendran S., Subramanian N., Seneviratne G., Zavahir J.S. (2021). Metal stress impacting plant growth in contaminated soil is alleviated by microbial siderophores. Role of Microbial Communities for Sustainability. Microorganisms for Sustainability.

[47] Lee S., Kang D.-W., Yoo J.-H., Park S.-W., Oh K.-S., Lee J.-H., Cho I.K., Moon B.-C., Kim W.-I. (2017). Determination of bioconcentration factor of heavy metal (loid)s in rice grown on soils vulnerable to heavy metal (loid)s contamination. Korean J. Soil. Sci. Fert..

[48] Raskin I., Smith R.D., Salt D.E. (1997). Phytoremediation of metals: Using plants to remove pollutants from the environment. Curr. Opin. Biotechnol..

[49] Nyquist J., Greger M. (2007). Uptake of Zn, Cu, and Cd in metal loaded Elodea canadensis. Environ. Exp. Bot..

[50] Greger M. (1999). Metal availability and bioconcentration in plants. Heavy Metal Stress in Plants.

[51] Gallego S.M., Benavides M.P., Tomaro M.L. (1996). Effect of heavy metal ion excess on sunflower leaves: Evidence for involvement of oxidative stress. Plant Sci..

[52] Salt D.E., Blaylock M., Kumar N.P., Dushenkov V., Ensley B.D., Chet I., Raskin I. (1995). Phytoremediation: A novel strategy for the removal of toxic metals from the environment using plants. Bio/Technology.

[53] Maurya P.K., Malik D., Yadav K.K., Kumar A., Kumar S., Kamyab H. (2019). Bioaccumulation and potential sources of heavy metal contamination in fish species in River Ganga basin: Possible human health risks evaluation. Toxicol. Rep..

[54] Carolin C.F., Kumar P.S., Saravanan A., Joshiba G.J., Naushad M. (2017). Efficient techniques for the removal of toxic heavy metals from aquatic environment: A review. J. Environ. Chem. Eng..

[55] Rai P.K., Lee S.S., Zhang M., Tsang Y.F., Kim K.-H. (2019). Heavy metals in food crops: Health risks, fate, mechanisms, and management. Environ. Int..

[56] Laskowski R. (1991). Are the top carnivores endangered by heavy metal biomagnification?. Oikos.

[57] Neeratanaphan L., Khamma S., Benchawattananon R., Ruchuwararak P., Appamaraka S., Intamat S. (2017). Heavy metal accumulation in rice (*Oryza sativa*) near electronic waste dumps and related human health risk assessment. Hum. Ecol. Risk Assess. Int. J..

[58] Fan Y., Li Y., Li H., Cheng F. (2018). Evaluating heavy metal accumulation and potential risks in soil-plant systems applied with magnesium slag-based fertilizer. Chemosphere.

[59] Edelstein M., Ben-Hur M. (2018). Heavy metals and metalloids: Sources, risks and strategies to reduce their accumulation in horticultural crops. Sci. Hortic..

[60] Masindi V., Muedi K.L. (2018). Environmental contamination by heavy metals. Heavy Met..

[61] Ali H., Khan E. (2019). Trophic transfer, bioaccumulation, and biomagnification of non-essential hazardous heavy metals and metalloids in food chains/webs—Concepts and implications for wildlife and human health. Hum. Ecol. Risk Assess. Int. J..

[62] Peralta-Videa J.R., Lopez M.L., Narayan M., Saupe G., Gardea-Torresdey J. (2009). The biochemistry of environmental heavy metal uptake by plants: Implications for the food chain. Int. J. Biochem. Cell Biol..

[63] FAO/WHO (2007). Evaluation of Certain Food Additives and Contaminants: Sixty-Eighth Report of the Joint FAO/WHO Expert Committee on Food Additives.

[64] Annex I. (2010). Report of the Tenth Meeting of the OIE Animal Production Food Safety Working Group.

[65] Robinson B., Duwig C., Bolan N., Kannathasan M., Saravanan A. (2003). Uptake of arsenic by New Zealand watercress (*Lepidium sativum*). Sci. Total Environ..

[66] Liu W.-X., Shen L.-F., Liu J.-W., Wang Y.-W., Li S.-R. (2007). Uptake of toxic heavy metals by rice (*Oryza sativa* L.) cultivated in the agricultural soil near Zhengzhou City, People’s Republic of China. Bull. Environ. Contam. Toxicol..

[67] Mao C., Song Y., Chen L., Ji J., Li J., Yuan X., Yang Z., Ayoko G.A., Frost R.L., Theiss F. (2019). Human health risks of heavy metals in paddy rice based on transfer characteristics of heavy metals from soil to rice. Catena.

[68] Song T., An Y., Cui G., Tong S., He J. (2021). Bioconcentrations and health risk assessment of heavy metals in crops in the Naoli River Basin agricultural area, Sanjiang Plain, China. Environ. Earth Sci..

[69] Chary N.S., Kamala C., Raj D.S.S. (2008). Assessing risk of heavy metals from consuming food grown on sewage irrigated soils and food chain transfer. Ecotoxicol. Environ. Saf..

[70] Tang L., Luo W.J., He Z.L., Gurajala H.K., Hamid Y., Khan K.Y., Yang X.E. (2018). Variations in cadmium and nitrate co-accumulation among water spinach genotypes and implications for screening safe genotypes for human consumption. J. Zhejiang Univ. Sci. B.

[71] Otitoju O., Otitoju G., Iyeghe L., Onwurah I. (2014). Quantification of heavy metals in some locally produced rice (*Oryza sativa*) from the northern region of Nigeria. J. Environ. Earth Sci..

[72] Ng C.C., Rahman M.M., Boyce A.N., Abas M.R. (2016). Heavy metals phyto-assessment in commonly grown vegetables: Water spinach (*I. aquatica*) and okra (*A. esculentus*). SpringerPlus.

[73] Leitenmaier B., Küpper H. (2013). Compartmentation and complexation of metals in hyperaccumulator plants. Front. Plant Sci..

[74] Nazir R., Khan M., Masab M., Rehman H.U., Rauf N.U., Shahab S., Ameer N., Sajed M., Ullah M., Rafeeq M. (2015). Accumulation of heavy metals (Ni, Cu, Cd, Cr, Pb, Zn, Fe) in the soil, water and plants and analysis of physico-chemical parameters of soil and water collected from Tanda Dam Kohat. J. Pharm. Sci. Res..

[75] Gladyshev M., Gribovskaya I., Ivanova E., Moskvichova A., Muchkina E.Y., Chuprov S. (2001). Metal concentrations in the ecosystem and around recreational and fish-breeding pond Bugach. Water Resour..

[76] Hapke H.-J. (1996). Heavy metal transfer in the food chain to humans. Fertilizers and Environment. Developments in Plant and Soil Sciences.

[77] Kasiulienė A., Paulauskas V., Marozas V., Waara S. (2019). Accumulation of heavy metals in forest dwarf shrubs and dominant mosses as bioindicators of atmospheric pollution. J. Elem. Olszt. Pol. Tow. Magnezol..

[78] Windham-Myers L., Fleck J.A., Ackerman J.T., Marvin-DiPasquale M., Stricker C.A., Heim W.A., Bachand P.A., Eagles-Smith C.A., Gill G., Stephenson M. (2014). Mercury cycling in agricultural and managed wetlands: A synthesis of methylmercury production, hydrologic export, and bioaccumulation from an integrated field study. Sci. Total Environ..

[79] Rahman Z., Singh V.P. (2019). The relative impact of toxic heavy metals (THMs) (arsenic (As), cadmium (Cd), chromium (Cr)(VI), mercury (Hg), and lead (Pb)) on the total environment: An overview. Environ. Monit. Assess..

[80] Yu Z., Gunn L., Wall P., Fanning S. (2017). Antimicrobial resistance and its association with tolerance to heavy metals in agriculture production. Food Microbiol..

[81] Fu Z., Xi S. (2020). The effects of heavy metals on human metabolism. Toxicol. Mech. Methods.

[82] Huang Y., Wang L., Wang W., Li T., He Z., Yang X. (2019). Current status of agricultural soil pollution by heavy metals in China: A meta-analysis. Sci. Total Environ..

[83] Engwa G.A., Ferdinand P.U., Nwalo F.N., Unachukwu M.N. (2019). Mechanism and health effects of heavy metal toxicity in humans. Poisoning in the Modern World-New Tricks for an Old Dog.

[84] Ugulu I., Ahmad K., Khan Z.I., Munir M., Wajid K., Bashir H. (2021). Effects of organic and chemical fertilizers on the growth, heavy metal/metalloid accumulation, and human health risk of wheat (*Triticum aestivum* L.). Environ. Sci. Pollut. Res..

[85] Vardhan K.H., Kumar P.S., Panda R.C. (2019). A review on heavy metal pollution, toxicity and remedial measures: Current trends and future perspectives. J. Mol. Liq..

[86] Izah S.C., Chakrabarty N., Srivastav A.L. (2016). A review on heavy metal concentration in potable water sources in Nigeria: Human health effects and mitigating measures. Expos. Health.

[87] Vigneri R., Malandrino P., Gianì F., Russo M., Vigneri P. (2017). Heavy metals in the volcanic environment and thyroid cancer. Mol. Cell. Endocrinol..

[88] Sabath E., Robles-Osorio M.L. (2012). Renal health and the environment: Heavy metal nephrotoxicity. Nefrología.

[89] Dapul H., Laraque D. (2014). Lead poisoning in children. Adv. Pediatr..

[90] Seidler A., Hellenbrand W., Robra B.-P., Vieregge P., Nischan P., Joerg J., Oertel W., Ulm G., Schneider E. (1996). Possible environmental, occupational, and other etiologic factors for Parkinson’s disease: A case-control study in Germany. Neurology.

[91] Rahman M.M., Owens G., Naidu R. (2009). Arsenic levels in rice grain and assessment of daily dietary intake of arsenic from rice in arsenic-contaminated regions of Bangladesh—Implications to groundwater irrigation. Environ. Geochem. Health.

[92] Oberoi S., Barchowsky A., Wu F. (2014). The global burden of disease for skin, lung, and bladder cancer caused by arsenic in food. Cancer Epidemiol. Prev. Biomark..

[93] Mandal P. (2017). An insight of environmental contamination of arsenic on animal health. Emerg. Contam..

[94] Karagas M.R., Punshon T., Davis M., Bulka C.M., Slaughter F., Karalis D., Argos M., Ahsan H. (2019). Rice intake and emerging concerns on arsenic in rice: A review of the human evidence and methodologic challenges. Curr. Environ. Health Rep..

[95] Ötleş S., Çağındı Ö. (2010). Health importance of arsenic in drinking water and food. Environ. Geochem. Health.

[96] Jomova K., Jenisova Z., Feszterova M., Baros S., Liska J., Hudecova D., Rhodes C., Valko M. (2011). Arsenic: Toxicity, oxidative stress and human disease. J. Appl. Toxicol..

[97] Rahimzadeh M.R., Rahimzadeh M.R., Kazemi S., Moghadamnia A.A. (2017). Cadmium toxicity and treatment: An update. Casp. J. Intern. Med..

[98] Himeno S., Aoshima K. (2019). Cadmium Toxicity: New Aspects in Human Disease, Rice Contamination, and Cytotoxicity.

[99] Dattilo A.M., Miguel S.G. (2003). Chromium in health and disease. Nut. Today.

[100] Onakpa M.M., Njan A.A., Kalu O.C. (2018). A review of heavy metal contamination of food crops in Nigeria. Ann. Glob. Health.

[101] Papanikolaou N.C., Hatzidaki E.G., Belivanis S., Tzanakakis G.N., Tsatsakis A.M. (2005). Lead toxicity update. A brief review. Med. Sci. Monit..

[102] Wani A., Ara A., Usmani J. (2015). Lead toxicity: A review. Interdiscipl. Toxicol..

[103] Zahir F., Rizwi S.J., Haq S.K., Khan R.H. (2005). Low dose mercury toxicity and human health. Environ. Toxicol. Pharmacol..

[104] Mousavi A., Chávez R.D., Ali A.-M.S., Cabaniss S.E. (2011). Mercury in natural waters: A mini-review. Environ. Forensics.

[105] Tóth G., Hermann T., Da Silva M., Montanarella L. (2016). Heavy metals in agricultural soils of the European Union with implications for food safety. Environ. Int..

[106] Chin N.P. (2010). Environmental toxins: Physical, social, and emotional. Breastfeed. Med..

[107] Bhunia P. (2017). Environmental Toxicants and Hazardous Contaminants: Recent Advances in Technologies for Sustainable Development. J. Hazard. Toxic Radioact. Waste.

[108] Cho-Ruk K., Kurukote J., Supprung P., Vetayasuporn S. (2006). Perennial plants in the phytoremediation of lead-contaminated soils. Biotechnology.

[109] Tangahu B.V., Sheikh Abdullah S.R., Basri H., Idris M., Anuar N., Mukhlisin M. (2011). A review on heavy metals (As, Pb, and Hg) uptake by plants through phytoremediation. Int. J. Chem. Eng..

[110] Kumar A., Prasad M., Achary V.M.M., Panda B.B. (2013). Elucidation of lead-induced oxidative stress in Talinum triangulare roots by analysis of antioxidant responses and DNA damage at cellular level. Environ. Sci. Pollut. Res..

[111] Gichner T., Žnidar I., Száková J. (2008). Evaluation of DNA damage and mutagenicity induced by lead in tobacco plants. Mut. Res. Gen. Toxicol. Environ. Mutagen..

[112] Sharma A., Sidhu G.P.S., Araniti F., Bali A.S., Shahzad B., Tripathi D.K., Brestic M., Skalicky M., Landi M. (2020). The role of salicylic acid in plants exposed to heavy metals. Molecules.

[113] Duxbury J.M., Panaullah G., Zavala Y.J., Loeppert R.H., Ahmed Z.U. (2009). Impact of use of As-contaminated groundwater on soil as content and paddy rice production in Bangladesh. Food Fertil. Technol. Cent. Tech Bull.

[114] Saha G.C., Ali M.A. (2007). Dynamics of arsenic in agricultural soils irrigated with arsenic contaminated groundwater in Bangladesh. Sci. Total Environ..

[115] Samal A.C., Kar S., Bhattacharya P., Santra S.C. (2011). Human exposure to arsenic through foodstuffs cultivated using arsenic contaminated groundwater in areas of West Bengal, India. J. Environ. Sci. Health Part A.

[116] Moulick D., Santra S.C., Ghosh D. (2018). Effect of selenium induced seed priming on arsenic accumulation in rice plant and subsequent transmission in human food chain. Ecotoxicol. Environ. Saf..

[117] Arunakumara K., Walpola B.C., Yoon M.-H. (2013). Current status of heavy metal contamination in Asia’s rice lands. Rev. Environ. Sci. Bio/Technol..

[118] Gimeno-García E., Andreu V., Boluda R. (1996). Heavy metals incidence in the application of inorganic fertilizers and pesticides to rice farming soils. Environ. Pollut..

[119] Liu Y., Li S., Ni Z., Qu M., Zhong D., Ye C., Tang F. (2016). Pesticides in persimmons, jujubes and soil from China: Residue levels, risk assessment and relationship between fruits and soils. Sci. Total Environ..

[120] Wickerham E.L., Lozoff B., Shao J., Kaciroti N., Xia Y., Meeker J.D. (2012). Reduced birth weight in relation to pesticide mixtures detected in cord blood of full-term infants. Environ. Int..

[121] Chen H., Zheng C., Tu C., Shen Z. (2000). Chemical methods and phytoremediation of soil contaminated with heavy metals. Chemosphere.

[122] Perera T.A., Tirimanne S., Seneviratne G., Zavahir J.S. (2021). Role of Microbial Communities in Sustainable Rice Cultivation. Role of Microbial Communities for Sustainability. Microorganisms for Sustainability.

[123] European Community (1986). Directive 86/276/EEC: Directive on the protection of the environment and in particular of soil, when sewage sludge is used in agriculture, Brussels. Off. J. Eur. Community.

[124] Rastetter N., Gerhardt A. (2017). Toxic potential of different types of sewage sludge as fertiliser in agriculture: Ecotoxicological effects on aquatic, sediment and soil indicator species. J. Soils Sediments.

[125] Wuana R.A., Okieimen F.E. (2011). Heavy metals in contaminated soils: A review of sources, chemistry, risks and best available strategies for remediation. Int. Sch. Res. Not..

[126] Bhagwat V.R. (2019). Safety of Water Used in Food Production. Food Safety and Human Health.

[127] Stoop W.A., Uphoff N., Kassam A. (2002). A review of agricultural research issues raised by the system of rice intensification (SRI) from Madagascar: Opportunities for improving farming systems for resource-poor farmers. Agric. Syst..

[128] Lawler S.P. (2001). Rice fields as temporary wetlands: A review. Isr. J. Zool..

[129] Roba C., Roşu C., Piştea I., Ozunu A., Baciu C. (2016). Heavy metal content in vegetables and fruits cultivated in Baia Mare mining area (Romania) and health risk assessment. Environ. Sci. Pollut. Res..

[130] Chaturvedi A.D., Pal D., Penta S., Kumar A. (2015). Ecotoxic heavy metals transformation by bacteria and fungi in aquatic ecosystem. World J. Microbiol. Biotechnol..

[131] Hejna M., Gottardo D., Baldi A., Dell’Orto V., Cheli F., Zaninelli M., Rossi L. (2018). Nutritional ecology of heavy metals. Animal.

[132] Zwolak A., Sarzyńska M., Szpyrka E., Stawarczyk K. (2019). Sources of soil pollution by heavy metals and their accumulation in vegetables: A review. Water Air Soil Pollut..

[133] Sumiahadi A., Acar R. (2018). A review of phytoremediation technology: Heavy metals uptake by plants. IOP Conference Series: Earth and Environmental Science, Proceedings of the 4th International Conference on Sustainable Agriculture and Environment (4th ICSAE) Surakarta, Indonesia, 10–12 August 2017.

[134] Fatima F., Hashim A., Anees S. (2021). Efficacy of nanoparticles as nanofertilizer production: A review. Environ. Sci. Pollut. Res..

[135] Gu H.-H., Qiu H., Tian T., Zhan S.-S., Chaney R.L., Wang S.-Z., Tang Y.-T., Morel J.-L., Qiu R.-L. (2011). Mitigation effects of silicon rich amendments on heavy metal accumulation in rice (*Oryza sativa* L.) planted on multi-metal contaminated acidic soil. Chemosphere.

[136] Mulligan C., Yong R., Gibbs B. (2001). Remediation technologies for metal-contaminated soils and groundwater: An evaluation. Eng. Geol..

[137] Dermont G., Bergeron M., Richer-Laflèche M., Mercier G. (2010). Remediation of metal-contaminated urban soil using flotation technique. Sci. Total Environ..

[138] Zhao X.-l., Saigusa M. (2007). Fractionation and solubility of cadmium in paddy soils amended with porous hydrated calcium silicate. J. Environ. Sci..

[139] Geebelen W., Sappin-Didier V., Ruttens A., Carleer R., Yperman J., Bongué-Boma K., Mench M., Van der Lelie N., Vangronsveld J. (2006). Evaluation of cyclonic ash, commercial Na-silicates, lime and phosphoric acid for metal immobilisation purposes in contaminated soils in Flanders (Belgium). Environ. Pollut..

[140] Chen X.-X., Liu Y.-M., Zhao Q.-Y., Cao W.-Q., Chen X.-P., Zou C.-Q. (2020). Health risk assessment associated with heavy metal accumulation in wheat after long-term phosphorus fertilizer application. Environ. Pollut..

